# A novel dominant glossy mutation causes suppression of wax biosynthesis pathway and deficiency of cuticular wax in *Brassica napus*

**DOI:** 10.1186/1471-2229-13-215

**Published:** 2013-12-14

**Authors:** Yuanyuan Pu, Jie Gao, Yanli Guo, Tingting Liu, Lixia Zhu, Ping Xu, Bin Yi, Jing Wen, Jinxing Tu, Chaozhi Ma, Tingdong Fu, Jitao Zou, Jinxiong Shen

**Affiliations:** 1 National Key Laboratory of Crop Genetic Improvement, Huazhong Agricultural University, Wuhan 430070, China; 2 National Research Council Canada, Saskatoon, Saskatchewan S7N 0 W9, Canada

**Keywords:** *Brassica napus*, Glossy mutant, Genetic mapping, Wax biosynthesis, Microarray assays, Candidate genes

## Abstract

**Background:**

The aerial parts of land plants are covered with cuticular waxes that limit non-stomatal water loss and gaseous exchange, and protect plants from ultraviolet radiation and pathogen attack. This is the first report on the characterization and genetic mapping of a novel dominant glossy mutant (*BnaA.GL*) in *Brassica napus*.

**Results:**

Transmission electron microscopy revealed that the cuticle ultrastructure of GL mutant leaf and stem were altered dramatically compared with that of wide type (WT). Scanning electron microscopy corroborated the reduction of wax on the leaf and stem surface. A cuticular wax analysis of the GL mutant leaves further confirmed the drastic decrease in the total wax content, and a wax compositional analysis revealed an increase in aldehydes but a severe decrease in alkanes, ketones and secondary alcohols. These results suggested a likely blockage of the decarbonylation step in the wax biosynthesis pathway. Genetic mapping narrowed the location of the *BnaA.GL* gene to the end of A9 chromosome. A single-nucleotide polymorphism (SNP) chip assay in combination with bulk segregant analysis (BSA) also located SNPs in the same region. Two SNPs, two single sequence repeat (SSR) markers and one IP marker were located on the flanking region of the *BnaA.GL* gene at a distance of 0.6 cM. A gene homologous to *ECERIFERUM1* (*CER1*) was located in the mapped region. A cDNA microarray chip assay revealed coordinated down regulation of genes encoding enzymes of the cuticular wax biosynthetic pathway in the glossy mutant, with *BnCER1* being one of the most severely suppressed genes.

**Conclusions:**

Our results indicated that surface wax biosynthesis is broadly affected in the glossy mutant due to the suppression of the *BnCER1* and other wax-related genes. These findings offer novel clues for elucidating the molecular basis of the glossy phenotype.

## Background

The plant cuticle acts as a hydrophobic layer to protect land plants against biotic and abiotic stresses. The cuticle layer is primarily composed of polymeric cutin and lipidic cuticular wax [1,2]; the cutin polymer is the framework, and waxes are interspersed within the cutin. Cuticular wax provides a protective barrier to biotic and abiotic stresses, including drought, pests, pathogens and UV radiation [3,4], and defects result in perturbed cuticle permeability. Cuticular wax biosynthesis is regulated in response to drought, reducing water loss rates in some plants [5-7]. *Arabidopsis*[8-10], rice [11,12] and tomato [13,14] mutants with wax alterations display sparse crystals on the surface of aerial organs and enhanced sensitivity to drought. Deficiency in cuticular wax has also been correlated with organ fusions and reduced fertility [8,15,16].

The aliphatic constituents of waxes are derived from saturated very long chain fatty acid. Fatty acids with chain length of up to C16 and C18 are synthesized in the plastids and subsequently exported to the cytoplasm where they are further elongated to very long-chain fatty acids (VLCFAs; C20 to C34) through the sequential addition of two-carbon units in a reaction catalyzed by fatty acid elongase complexes in the endoplasmic reticulum [1,2]. Cuticular waxes contain species-specific compound classes and carbon chain length patterns [1,2]. In *Arabidopsis*, the cuticular wax of stems consists of VLCFA alkanes, aldehydes, fatty acids, primary alcohols, wax esters, secondary alcohols and ketones, with 80-90% alkanes, secondary alcohols and ketones [17,18]. The biosynthesis of these compounds involves two pathways: the acyl-reduction pathway in which primary alcohols and wax esters are synthesized and the decarbonylation pathway through which aldehydes, alkanes, ketones and secondary alcohols are synthesized [18].

In *Arabidopsis*, the *cer1-1* mutant is characterized by a drastic decrease in the products of the alkane-forming pathway (alkanes, secondary alcohols and ketones) and a corresponding increase in aldehydes [19-21]. The product of the *ECERIFERUM* (*CER1*) functions as a putative aldehyde decarbonylase [19,20], and it was recently shown that *CER1* interacts with the wax-associated gene *CER3* and cytochrome b5 isoforms (*CYTB5s*) [22]. *CER1* homologs are present in both dicot and monocot species and are structurally conserved. Additionally, all *CER1* proteins contain iron-binding (histidine-rich) motifs, suggesting that *CER1* homologous proteins have a similar function among organisms [19].

Normally, the waxless character is controlled by recessive genes. To our knowledge, there have been few reports of dominant mutations. In wheat, the inheritance of glaucousness is mainly governed by two sets of dominant genes, *W1* and *W2*, which promote a glaucous phenotype, and *Iw1* and *Iw2*, which inhibit it. *W1* and *Iw1* are located on the short arm of chromosome 2B (2BS) and *W2* and *Iw2* on 2DS [23]. Recently, a genetic approach with the detailed biochemical characterization of wax compounds was used to characterize the *Iw1* locus, which inhibits the formation of β- and hydroxy-β-diketones in the peduncle and flag leaf blade cuticles. This inhibitory effect was found to be independent of the genetic background or tissue and was accompanied by minor but consistent increases in n-alkanes and C24 primary alcohols [24]. In banana, the ratio of waxy versus nonwaxy pseudostem in the cross SH-3362 × Long Tavoy was 1:1 (x^2^ = 0.22), indicating that the genotypes of SH-3362 and Long Tavoy should be *wxwx* and *Wxwx*, respectively. The F_1_ obtained by crossing FR (slight pseudostem waxiness) × C4 (nonwaxy pseudostem) were glossy, and the F_2_ segregated for this trait (glossy: wax = 3:1, x^2^ = 1.15) indicating that the nonwaxy pseudostem may be a single dominant in this population, and that the genotype of C4 should be *WxWx*. But in other segregating populations the situation was more complex, suggesting that genetic modifiers can overcome the action of the *Wx* allele [25].

Although wax-deficient mutants have been isolated in a number of plant species [26,27], there are few reports focused on the study of cuticular wax in *Brassica* species [28,29]. Since 1990, breeders have focused on the genetic analysis of the wax-less phenotype and have suggested that this feature can be used as a morphological marker in hybrid breeding. Recently, in *Brassica rapa*, the *BrWax1* (*Brassica Wax*) gene was located to a 86.4 kb genomic DNA fragment on linkage group A1. *Bra013809*, the homologous gene of *CER2* was speculated to be the candidate gene [30].

To our knowledge, this is the first report on the characterization and genetic mapping of a dominant glossy mutant (*BnaA.GL*) in *B. napus*. The *GL* mutant exhibited a drastic decrease in wax and enhanced cuticle permeability. Linkage of SNP markers to the *BnaA.GL* gene were achieved using an SNP chip assay combined with BSA, and the *BnaA.GL* gene was localized to the end of the *B. napus* A9 chromosome. The *B. napus GL* mutant was found to share biochemical characteristics of the *cer1-1* mutant in *Arabidopsis thaliana*. Additionally, a gene homologous to *CER1* was found to be located in the candidate region. A cDNA microarray assay showed that the expression of a group of genes encoding cuticular wax biosynthetic enzymes and fatty acid synthesis were down-regulated in the leaf tissues of the glossy mutant. We found that the *BnCER1* gene was dramatically suppressed in the *GL* mutant but no mutation affecting the function of the *BnCER1* gene was obvious. We conclude that the *BnaA.GL* mutation caused down-regulation of cuticular wax biosynthetic genes, particularly the *CER1* gene. Since the *BnaA.GL* allele behaves in a dominant fashion in regulating wax biosynthesis, this glossy mutant is unique from other *cer* mutants reported to date and may prove useful for future study on the regulation of wax biosynthesis pathways in plants.

## Result

### Identification of the *GL* mutant

The *GL* mutant (6–1025) was initially discovered from a large breeding population. In this project line 6–3476 was used as the wide type to cross with *GL* mutant 6–1025 to generate F_1_ seeds. One F_1_ plant derived from this cross was then used to develop a double haploid (DH) population through microspore culture as described in a previous report [31]. In these DH lines there was clear segregation in terms of the glossy phenotype. We selected line-7 (DH line-7) and line-69 (DH line-69) which both exhibited a distinctive glossy phenotype, and line-2 (DH line-2), and line-3 (DH line-3) that appeared normal for further study.

The F_1_ (or RF_1_) plants of reciprocal crosses between WT and the *GL* mutant were all glossy, indicating that the glossy trait was dominant. The BC_1_ progeny developed from the crosses between the F_1_ plant and WT displayed a 1:1 ratio of glossy to normal plants. Moreover, the glossy to normal phenotype ratio in the F_2_ population was approximately 3:1 (Table 1), indicating that this was a case in which one Mendelian locus controlled the glossy trait. The glossy gene was tentatively designated as the *BnaA.GL* gene.

**Table 1:** Segregation of glossy trait in the BC1 and F2 progenies of two crosses

**Combination**	**Population**	**No. of glossy**	**No. of wax**	**Expected ratio**	**P**
WT × mutant	BC1	128	117	1:1	0.48
	F2	278	113	3:1	0.08

### Abnormal epidermis formed and water permeability increased in the *GL* mutant

**Figure 1:**
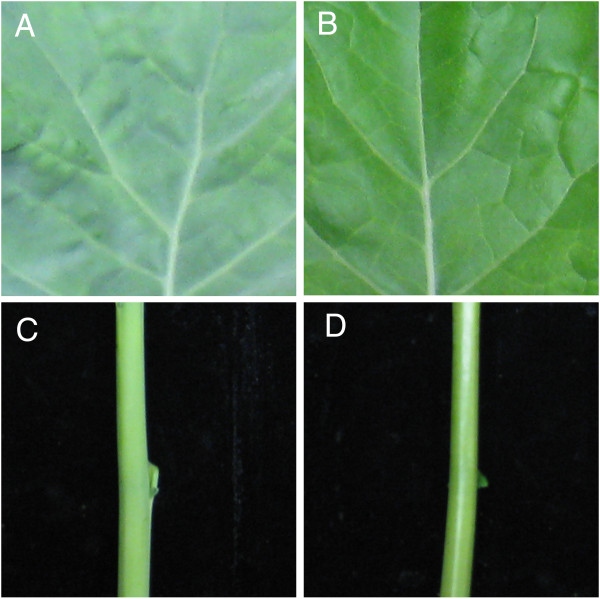
**Morphological characters of WT and the *GL *mutant.** The leaf **(A)** and stem **(C)** of WT appear glaucous, as compared to the glossy phenotype of the *GL* mutant leaf **(B)** and stem **(D)**.

**Figure 2:**
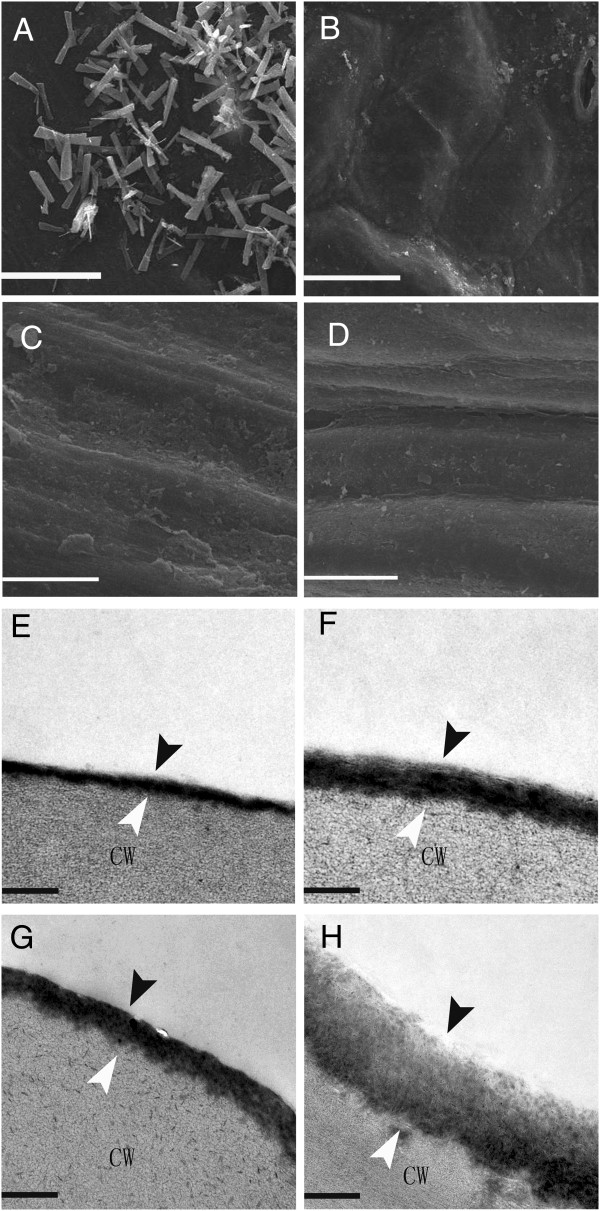
**Epicuticular wax and cuticle layer of WT and the *GL *mutant. (A-D)** Scanning electron microscopy. **(A)** Wax crystals on the WT leaf were dense, with high proportion of tubular-like wax crystals. **(B)** Wax crystals sparsely distributed on the leaf of the *GL* mutant. Bar =20 μm. **(C)** Wax crystals on the WT stem with a high proportion of plate-like wax crystals. **(B)** Fewer wax crystals were present on the stem of the *GL* mutant. Bar =10 μm. **(E-H)** Transmission electron microscopy. The leaf and stem cuticle proper is indicated by black arrowheads and the cuticle layer is indicated by white arrowheads. The cuticle ultrastructure of *GL* mutant leaf **(F)** and stem **(H)** were altered dramatically compared with that of WT leaf **(E)** and stem **(G)**. The cuticle membrane (cuticle proper plus cuticular layer) of *GL* leaf and stems was thicker, and less osmiophilic. Bar =200 nm.

The glossy phenotype of the *GL* mutant’s leaf and stem surface is shown in Figure 1B, D. Scanning electron microscopy (SEM) was also used to assess the density of wax crystals on leaf and stem tissue. This revealed a clear reduction of wax crystals in some regions of leaf surface (Figure 2A-D). Leaf and stem cross sections were further examined using transmission electron microscopy (TEM), which indicated that that the cuticle ultrastructure of *GL* mutant leaf (Figure 2F) and stem (Figure 2H) were altered dramatically compared with that of WT leaf (Figure 2E) and stem (Figure 2G). The cuticle membrane (cuticle proper plus cuticular layer) of *GL* leaf and stem were less osmiophilic, as indicated by the reduced electron density, but more thick compared with those of wild type, especially the stem.

**Figure 3:**
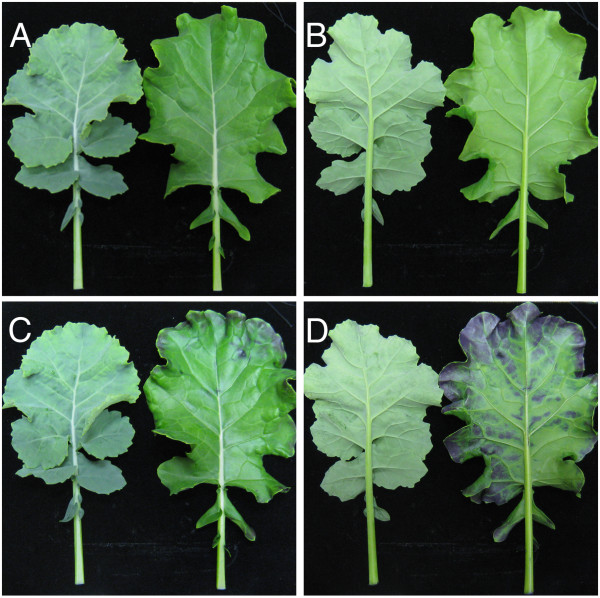
**Defective cuticle in the *GL *mutant.** Toluidine blue staining pattern of WT and the *GL* mutant. WT (left), mutant (right). Before staining **(A, B)**, after staining **(C, D)**.

A distorted cuticular layer often results in an increased permeability of leaves [32-34]. To test this, we incubated 4 weeks old leaves with Toluidine Blue (TB) solution (0.05% w/v) for 2 min, and assessed the staining intensities. As show in (Figure 3C, D left), WT leaves were barely stained whereas many parts of the mutant leaves were heavily stained (Figure 3C, D right). Furthermore, the *GL* mutant rapidly lost chlorophyll content and showed a significantly higher water loss rate when compared with WT (Additional file 1). These results were consistent with the observation that the cuticular layer of the leaf was abnormal, thus suggesting that the mutants were compromised in the strength of water permeability barrier.

### Decrease in total cuticular waxes and alteration of wax composition in the *GL* mutant

**Figure 4:**
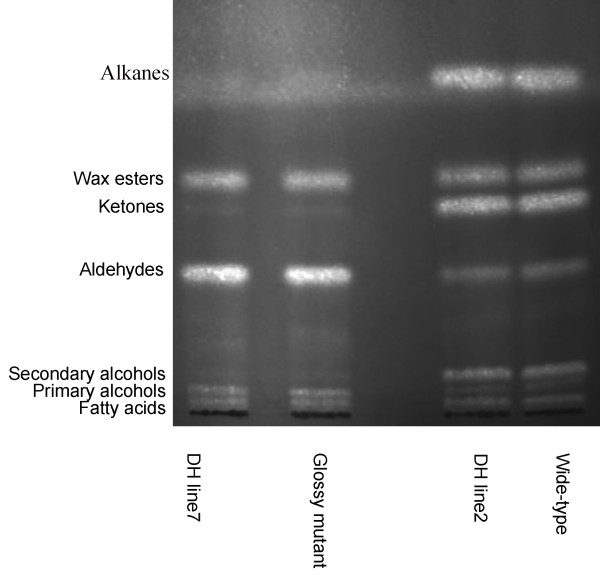
**Thin layer chromatography (TLC) of the leaf wax mixture.** The compound classes are labeled on the left. The different lanes show the separation of the wax extracts from WT, mutant and DH line leaves.

To dissect the changes in chemical components that were responsible for the glossy phenotype, we used thin-layer chromatography (TLC) for quantitative and compositional analyses of leaf wax extracts. TLC analysis revealed a distinctively different pattern of wax composition in the mutants (Figure 4). The *GL* mutant and DH line-7 had total wax coverage of 3.2 μg/cm^2^ and 6.4 μg/cm^2^, which represented a reduction of 89% and 77%, respectively, when compared with the wild type and DH line-2 (Table 2).

**Table 2:** Cuticular wax composition of wild-type, mutant and DH line

**Line**	**Total**	**Alkanes**	**Wax esters**	**Ketones**	**Aldehydes**	**Secondary alcohols**	**Fatty acids**	**Primary alcohols**
Wide-type	29.4 ± 0.4	16.2 ± 0.5	0.6 ± 0.2	7.8 ± 0.2	1.1 ± 0.5	2.8 ± 0.5	0.3 ± 0.1	0.7 ± 0.2
Glossy mutant	3.2 ± 0.4	-	0.1 ± 0.0	-	2.7 ± 0.4	-	0.2 ± 0.0	0.3 ± 0.0
DH line-2	27.4 ± 3.7	13.9 ± 0.4	0.7 ± 0.4	5.9 ± 2.3	1.7 ± 0.4	4.1 + 1.1	0.2 ± 0.1	0.8 ± 0.4
DH line-7	6.4 ± 2.0	-	0.3 ± 0.1	-	4.9 ± 1.3	-	0.7 ± 0.4	0.5 ± 0.4

Total wax content and coverage of individual compound classes (μg/cm^2^) were given as mean values ± SE (n = 3). “-” and 0.0 denote values that were below 0.05 μg/cm^2^.

Looking into different cuticular wax compound classes, alkanes, secondary alcohols (2° alcohols) and ketones were not detectable (or below detection limits) in the mutants (Figure 4; Table 2). We also detected reductions in both primary alcohols and wax esters. There was also a several fold reduction in wax esters, which were found at 0.1 μg/cm^2^ and 0.3 μg/cm^2^ in the *GL* mutant and DH line-7, respectively, while the WT and the DH line-2 had wax esters at 0.6 μg/cm^2^ and 0.7 μg/cm^2^. Primary alcohols were found at 0.3 μg/cm^2^ and 0.5 μg/cm^2^ in the *GL* mutant and DH line-7, respectively, while the WT and the DH line-2 had levels of at 0.7 μg/cm^2^ and 0.8 μg/cm^2^, respectively (Table 2). Against the backdrop of reduction in all these compound classes, our analysis revealed clear increases in the content of the aldehydes, which in both the *GL* mutant and DH line-2 were at approximately 3 fold of that of the WT (Table 2).

**Figure 5:**
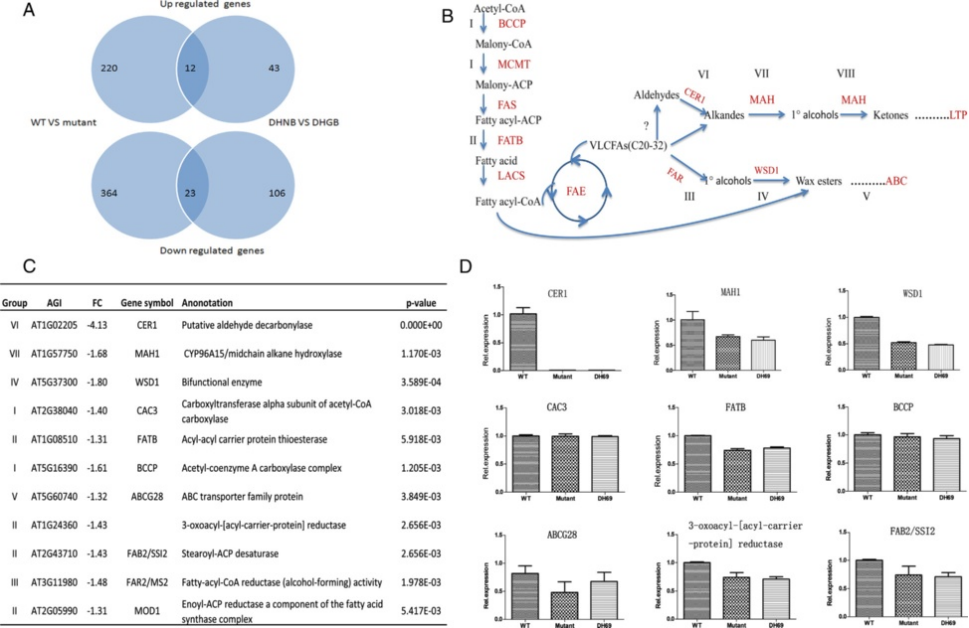
**Downregulation of cuticular wax biosynthetic genes in the glossy mutant. (A)** Venn diagrams showing the overlapping and specific DEGs in the WT vs. mutant and DHNB vs. DHGB analyses. **(B)** Simplified cuticular wax biosynthetic pathway, as adapted from previous reports [2,7,27]. The numbers indicate the mean fold change of the genes belonging to individual gene groups (I to VIII), as marked in **(B)**. The question marks and dotted lines indicate unidentified enzymes and processes, respectively. **(C)** List of the wax biosynthetic genes and fatty acid synthesis genes down-regulated in both the glossy mutant and glossy DH line 69. The P values were corrected for multiple testing using FDR methodology. The group of genes was classified based on their biochemical function. FC, fold change. **(D)** qRT-PCR of wax biosynthetic gene expression. Total RNA was extracted from 6-week-old leaves. The transcript levels were examined using qRT-PCR. The bars indicate SE of the mean.

Previous studies have shown that mutations in *CER1* block the conversion of stem wax C30 aldehydes (triacontanal) to C29 alkanes (nonacosane), and the secondary alcohols and ketones were also reduced in the mutants [19,20]. Because alkanes, secondary alcohols and ketones are major components of total wax, the lack of these three components would be expected to negatively impact the overall wax coverage [17,18]. Furthermore, the accumulation of aldehydes was also consistent with the deficiency of the CER1 step that converts aldehyde to alkandes in the *GL* mutant (Figure 4; Table 1; Figure 5).

### Fine mapping of the *BnaA.GL* gene

**Figure 6:**
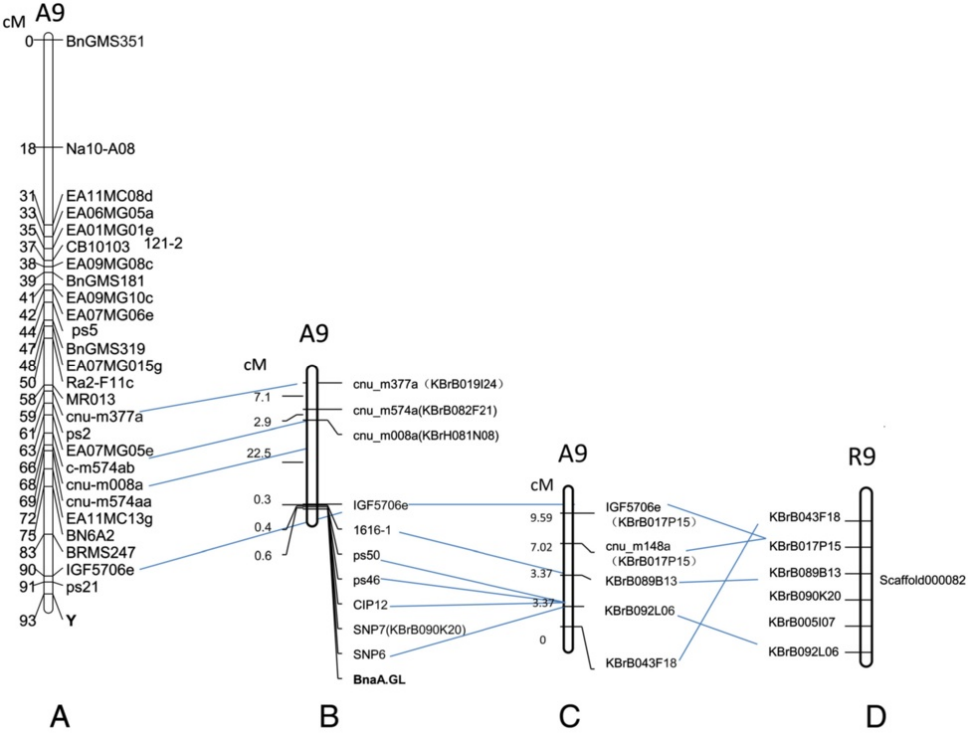
**Partial linkage maps of *B. napus *indicating the relative location of the *BnaA.GL *gene on linkage group A9. (A)** A linkage map of the region surrounding the *BnaA.GL* gene from the F_2_ population. **(B)** Partial linkage maps of *B. napus* indicate the relative location of the *BnaA.GL* gene on linkage group A9. **(C)** The partial A9 linkage map of the Tapidor-Ningyou Double Haploid (TNDH) linkage map of *B. napus*[38]. **(D)** Partial R9 (Scaffold000082) physical map of *B. rapa*[36,37].

**Figure 7:**
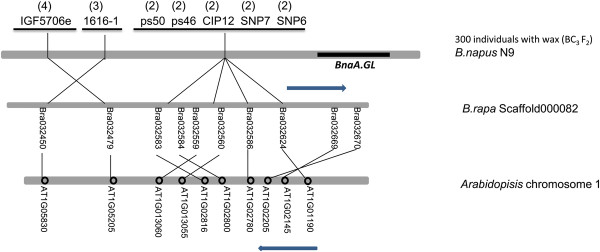
**Comparative mapping of the *BnaA.GL *gene.** Comparative analysis of the mapping region showing *B. rapa* genes and *Arabidopsis* genes [37,39].

Our initial mapping using the glossy leaf phenotype as a morphological marker (named Y) mapped the genetic lesion to linkage group A9 from an F_2_ population derived from a cross between WT and the glossy mutant. SSR and AFLP markers were assembled into a genetic linkage map with JOINMAP 3.0 (Figure 6A). We then developed seven amplified fragment-length polymorphism (AFLP) markers linked to the *BnaA.GL* gene using an AFLP assay in combination with BSA [35]. Only two of the markers were converted into useful sequence-characterized amplified region (SCAR) markers, and the SCAR marker 1616–1 was detected in the flanking region of the *BnaA.GL* gene (Figure 6B). Additional SSR markers were pursued based on a bacterial artificial chromosome (BAC) sequence close to the end of the *B. rapa* R9 chromosome (Figure 6D), resulting in two more SSR markers mapped to the flanking region of the *BnaA.GL* gene (Figure 6B) [36,37]. Since the BAC “KBrB043F18” was located on the very end of A9 [38] (Figure 6C), we developed markers based on the BAC sequences, but no molecular markers with polymorphism were detected based on the sequence of BAC “KBrB043F18” (Figure 6B, C, D). IP markers were designed based on a previous report [39], and one IP marker CIP12 was detected in the flanking region of the *BnaA.GL* gene (Figure 6B). Based on 300 normal individuals from a BC_3_F_2_ population (1200), all markers were used to survey the population. After genotype testing of all individuals in the mapping population, 4 individuals displaying recombination between *BnaA.GL* and IGF5706e were identified. The distance between IGF5706e and *BnaA.GL* was 1.33 cM. Among the 4 identified recombinants, 3 of the individuals displayed recombination between the *BnaA.GL* gene and 1616–1, and the distance between 1616–1 and *BnaA.GL* was 1 cM. In addition, 2 recombinants displayed recombination between *BnaA.GL* and the other SSR and IP markers, the distance between these markers and the *BnaA.GL* gene was 0.67 cM. All markers were on the same side of the *BnaA.GL* gene. These markers were mapped to a region of 0.67-1.33 cM in the flanking region of the *BnaA.GL* gene. Unfortunately, we failed to find any markers on the other side of the *BnaA.GL* gene. Compared to *B. rapa*, there appears to be an inversion at the end of the *B. napus* chromosome [38] (Figure 6B, C, D). All markers linked to the gene were used to compare the micro-colinearity of the regions flanking the genes with *B. rapa* and *Arabidopsis* (Figure 7).

### Transcriptome analysis uncovered different expression pattern in the mutants

In search of clues underlying the molecular basis of the glossy phenotype, we then conducted a microarray analysis using RNA from leaf tissue of similar developmental stages and performed a comparison of the global transcript level between WT vs. the *GL* mutant and double haploid normal line bulk (DHNB) segregates vs. DH glossy line bulk (DHGB) segregates. In the wild type vs. *GL* mutant analysis, 619 genes were detected as DEGs (differentially expressed genes): 232 DEGs showed a higher expression level in wild type, whereas 387 DEGs showed a higher expression level in the *GL* mutant (Figure 5A; Additional file 2). A total of 184 genes were determined to be DEGs in the analysis between the bulked glossy DH lines and the bulked normal DH lines (DHNB vs. DHGB), with 55 showing a higher expression level in DHNB and 129 showing a higher level in DHGB (Figure 5A; Additional file 2). We could identify 55 shared genes showing differential expression profiles through both sets of microarray experiments, among which 12 genes were more highly expressed in normal plants, and 23 genes were more highly expressed in glossy plants (Figure 5A; Additional file 2). Despite the fact that the phenotype was distinguished based on wax content, the Gene Ontology (GO) term categorization for both comparisons indicated that the majority of genes indentified are related to stress responses and to abiotic or biotic stimuli (Additional file 2; Additional file 3). For the genes related to lipid metabolism, only 29 genes were up-regulated and same number of genes were down-regulated in the wild type vs. *GL* mutant analysis; in DHNB vs. DHGB analysis, the number of up- and down-regulated genes were 11 and 5 (Additional file 3).

### Wax biosynthetic genes are down-regulated in the glossy mutant

A major functional category of the down-regulated genes emerged from the DHNB vs. DHGB microarray analysis included those encoding a subset of genes related to the wax biosynthesis pathway (Figure 5). The aliphatic components of cuticular waxes are derived from saturated very-long-chain fatty acids [4]. The homolog of a gene encoding the biotin carboxyl carrier protein (BCCP), a component of the acetyl-CoA carboxylase that catalyzes the first committed step of fatty acid synthesis in the plastids, was down regulated in the *GL* mutant (Figure 5B, 5C). Similarly, the homolog of a gene encoding the 3-oxoacyl-ACP reductase of the fatty acid synthase was also expressed at a lower level. The homolog of gene FATB, the acyl-acyl carrier protein thioesterase primarily responsible for hydrolyzing saturated acyl-ACPs to release saturated fatty acids into the cytosolic compartment, was reduced as well. Collectively, these results suggest that, at least at the transcript level, enzymes involved in the production of saturated fatty acids and precursors of the aliphatic components of waxes were negatively regulated in the leaf tissues of the *GL* mutant.

After the production of very long chain fatty acyl-CoAs, the biosynthesis of cuticular wax proceeds through two main pathways: an acyl reduction pathway, which produces primary alcohols and wax esters, and a decarbonylation pathway that generates aldehydes, alkanes, secondary alcohols and ketones. The putative aldehyde decarbonylase, *CER1,* and the midchain alkane hydroxylase, *MAH1,* are involved in the decarbonylation branch of the wax biosynthesis [18-20], [40-42]. Homologs of both *CER1* and *MAH1* were found to be down regulated, suggesting that the decarbonylation pathway was suppressed. Reduction of transcript levels of the homolog of the bifunctional enzyme wax synthase (*WSD1*) gene [42] (Figure 5C) indicated that the acyl reduction pathway of wax biosynthesis was likely affected negatively as well in the *GL* mutant. The different expression patterns of these genes in the *GL* mutants were verifiable by quantitative real-time PCR (qRT-PCR) (Figure 5D).

**Figure 8:**
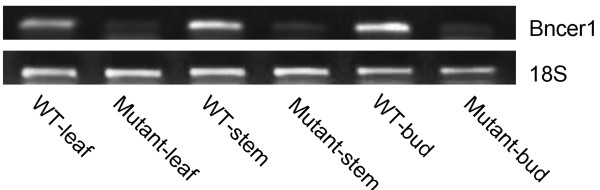
**Expression of the *BnCER1 *gene in WT and the *GL *mutant.** Semi-quantitative RT-PCR. RNA prepared from 4-week-old stems, leaves and buds. 18S rRNA was applied as a constitutively expressed control.

Among the differentially expressed genes found in the *GL* mutants, *BnCER1*, the homolog of *CER1* (At1g02205), was the most severely down-regulated. To investigate the expression patterns of *BnCER1* in different tissues of *B. napus* plants, RT-PCR (semi quantitative RT-PCR) was performed using RNA prepared from 4-week-old stems, leaves and buds. *BnCER1* was present at much higher levels in all the WT tissues examined (Figure 8), in accordance with a previously published report [20].

### *BnCER1* gene sequence analysis

**Figure 9:**
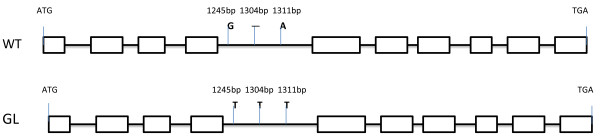
The major sequence differences are in intronic regions.

Based on the available genome information [37,43], there was approximately 250 kb between the closest marker SNP6 and *Bra032670* at the end of chromosome R9 in *B. rapa*. Although we failed to detect markers on the other side of the gene, the results suggested that this chromosome region could be a candidate for fine mapping and cloning of the gene. In light of the fact that *Bra032670* at the end of the chromosome was homologous to *CER1* (AT1G02205), the genetic lesion responsible for the glossy phenotype in *Arabidopsis*, we designed primers and amplified this gene sequence. It was found that the *Bra032670* gene has a length of 4.41 kb, with ten exons and nine introns. At least four copies of the *CER1* otholog gene were identified from the allotetraploid oil crop species *B. napus*. However, there was no apparent sequence alteration between WT and the *GL* mutant except in the fifth intron where three SNPs were identified (Figure 9; Additional file 4). Our results thus suggested that the *BnaA.GL* mutation directly resulted in the down-regulation of cuticular wax biosynthetic genes, particularly the *CER1* gene, leading to a deficiency of wax decarbonylation and, consequently, reduced wax deposition. Unlike previous reports in wax biosynthesis regulation, the *BnaA.GL* allele behaves in a dominant fashion.

## Discussion

### The decarbonylation pathway of wax biosynthesis was compromised in the *GL* mutant

The *GL* mutant features a glossy phenotype similar to the Arabidopsis *cer1-1* mutant. *CER1* was speculated to be an aldehyde decarbonylase, which catalyzes alkane biosynthesis through the decarboxylation pathway [19,20]. Data of wax composition analysis of the *GL* mutant also revealed biochemical characteristics resembling that of the *Arabidopsis cer1-1*[20]. Indeed, the wax compositional alterations were accentuated by decreases in the metabolites of the decarbonylation pathway, alkanes, secondary alcohols and ketones. There was also a conspicuous increase in proportions of aldehydes, the precursors of the metabolic step mediated by CER1. In comparison to the *Arabidopsis cer1* mutant, the deficiency of alkanes, secondary alcohols and ketones in the *GL* mutant was more extreme, consequently resulting in a dramatic reduction of total wax [17,18]. Thus, results of our biochemical analysis as a whole clearly indicate that the decarbonylation pathway of wax biosynthesis was compromised in the GL mutant, evidenced by the fact that alkanes, and the primary candidate gene for alkane synthesis *CER1*, were affected most.

### Mapping in *B. napus*

Our genetic analysis conclusively show that the glossy phenotype of the *GL* mutant is determined by a single Mendelian locus, *BnaA.GL.* But unlike the *cer1* mutant and all other wax biosynthesis mutants reported to date, the *BnaA.GL* locus acts in a dominant manner since reciprocal crosses between WT and the *GL* mutant were all glossy. We were able to map the genetic lesion to a linkage group close to the end of chromosome A9. Unfortunately, we failed to identify markers on the other side of the *BnaA.GL* gene, possibly due to the high level of relatedness of the parents. We are attempting to construct a larger population using a different line as the WT parent. This may allow us to reduce the interval covering the *BnaA.GL* gene, enabling us to predict the candidate genes within the region.

We found that the difference in the genetic linkage maps shown between Figure 6C to D was the location of BAC “KBrB043F18”. Compared to *B. rapa*, there appears to be an inversion at the end of the *B. napus* chromosome. We did not get polymorphic markers based on the sequence of the BAC “KBrB043F18”, possibly due to the genomic rearrangements on the corresponding region of A9.

All linked markers we detected were on one side of the *BnaA.GL* gene in *B. napus*, likely because the gene is located near the end of chromosome. The genome information of *B. rapa* has recently become available, and we made use of information on chromosome synteny between *Arabidopsis* and *B. rapa* to conduct genetic and physical comparative mapping of the *BnaA.GL* locus [37,43-49]. By comparative mapping with *B. rapa*, the distance from the closest marker and the last annotated gene on R9 is calculated as only 250 kb [43]. Markers linked to *BnaA.GL* were used to dissect the micro-colinearity of these regions in *Arabidopsis* and *B. rapa*. Moreover, SNP markers were developed using an SNP chip assay combined with BSA. This led to the detection of 36 SNPs (Additional file 5), 75% of which were located in the mapping region. In light of the finding that a homolog of *Arabidopsis CER1*, *Bra032670,* was located in the mapped region of the *BnaA.GL*, we then amplified and sequenced the *Bra032670* from the *GL* mutant. *Bra032670* shares 89% sequence identity in the coding DNA sequence (CDS) and 99% at the protein level with the *Arabidopsis* CER1. However, no apparent sequence alteration was found between WT and the *GL* mutant, except three SNPs in the fifth intron (Figure 9; Additional file 4).

### Wax biosynthetic genes are down-regulated in the glossy mutant

The reduced expression of multiple wax-related genes uncovered through microarray analysis, beginning from saturated fatty acid biosynthsis to the two branches of the wax biosynthesis pathway, provided a biochemical basis for the phenotype of reduced cuticular wax content. Furthermore, in accordance with the wax compositional changes of the *GL* mutants, *BnCER1* of the decarbonylation pathway of wax biosynthesis was singularly the most severely suppressed gene. Thus, our results show that although the gene *Bra032670* encoding *BnCER1* was not the genetic lesion in *GL* mutant, it nonetheless is the primary cause of compromised cuticular wax biosynthesis in the *GL* mutant. We suggest that the *BnaA.GL* gene acts as a regulatory factor that targets *BnCER1* and likely the wax biosynthesis pathway as a whole. This supposition is supported by the finding that the midchain alkane hydroxylase (*MAH1*) and a bifunctional enzyme (*WSD1*) were also down-regulated [41,42] in the *GL* mutant. *MAH1* is involved in the decarbonylation pathway, catalyzing the hydroxylation of alkanes to secondary alcohols [41]. Some steps of the fatty acid biosynthesis pathway, which is further upstream of the wax decarnylation pathway, were also impacted in the mutant leaf tissues [4]. It must be noted, however, that it is unclear whether the suppression of *CER1* resulted in the overall decrease in wax pathway genes or whether *CER1* is suppressed as a part of the overall down-regulation of the wax pathway, ultimately leading to a deficiency in wax decarbonylation and, consequently, reduced wax deposition.

## Conclusions

The *IW1*, *IW2* genes in wheat, and the *WxWx* gene in banana are dominant waxless character genes [23-25]. Except for these genes, however, no dominant glossy mutant has been reported to date. As distinguished from the *Arabidopsis cer* mutants reported thus far, the *BnaA.GL* allele behaves in a dominant fashion in regulating wax biosynthesis, although many details remain to be resolved. The glossy phenotype is a classic genetic marker trait in *Brassica* and could be used as a morphological marker in hybrid breeding. Molecular mapping and cloning of the *BnaA.GL* genes will also allow novel approaches for manipulating cuticle permeability to increase drought tolerance.

## Methods

### Plant materials and growth conditions

The *B. napus* glossy mutant 6–1025 (*BnaA.GL, *from Chengdu Academy of Agriculture and forestry Science) and WT 6–3476 were used in this study. The F_2_ population of 187 plants was derived from the WT × *GL* mutant cross. The F_1_ plants were backcrossed with WT for three generations to generate a BC_3_F_1_ population; the glossy individuals were selfed and the BC_3_F_2_ seeds were harvested. More than 1200 BC_3_F_2_ individuals were planted, and 300 individuals without a glossy phenotype were used for mapping purposes. The mapping populations were grown in the research field of Huazhong Agricultural University, Wuhan, People’ s Republic of China.

The plants used for the wax analysis, water permeability analysis and RNA extraction were grown in a soil-based compost under standard greenhouse conditions with a 16-h light/8-h dark cycle. The day/night temperature was 22/17°C (NRC Saskatoon, SK, Canada).

### SEM and TEM analyses

SEM was used to study the surface of leaves and stems of the mutant and WT plants. Fresh leaves and stems were collected after 4 weeks of growth and fixed overnight in 2% glutaraldehyde. The ensuing procedures were performed as previously reported [50].

For TEM analysis, fresh leaves and stems were collected from plants after 4 weeks of growth, and the tissue was fixed overnight in 2.5% (w/v) glutaraldehyde in 0.1 M phosphate buffer (pH 7.4). The ensuing procedures were performed according to a published report [50].

### Wax extraction and chemical characterization

Leaves were collected plants after 4 weeks of growth. Total cuticular wax mixtures were extracted by immersing the leaves in chloroform (CHCl_3_) twice for 30 s at room temperature. Equal-area round leaf disks were cut using a hole-puncher and used in the wax analysis. For the TLC analysis, the wax mixtures were separated on silica gel 60 using hexane-diethylether-acetic acid (90:7.5:1 [v/v/v]) and visualized by staining with primuline and UV light [41,42]. Each component was extracted from the silica gel, and n-tetracosane (C24 alkane) was added as an internal standard [41]. The solvent was subsequently evaporated under a gentle stream of nitrogen and treated with a mixture of 20 μL of bis-N, N-(trimethylsilyl) trifluoroacetamide (Sigma) in pyridine for 1 h at 70°C to convert all the waxes into trimethylsilyl derivatives. The wax composition was determined by capillary GC (6890 N; Agilent) and a mass spectrometric detector (5973 N; Agilent). The initial temperature of 50°C was held for 2 min, increased at 40°C/min to 200°C, held for 2 min at 200°C, increased again at 3°C/min to 320°C and held for 30 min at 320°C. Quantification was based on the flame ionization detector peak areas and the internal standard (C24). The total amount of cuticular wax was expressed per unit of leaf surface area, and the areas were determined by π. The molecular identities were determined using a GC Agilent 6890 and an Agilent 5973 mass spectrometric detector. The GC program used was according to previous reports [41].

### Mapping

DNA was extracted using a modified CTAB (cetyltrimethylammonium bromide) method [51]. For constructing a rough flanking map linked to the *BnaA.GL* gene, an F_2_ population consisting of 187 individuals was used. The glossy phenotype as a genetic marker (Y) was used, and a linkage map was constructed as previously reported [52]. A bulk segregant analysis (BSA) combined with the AFLP technique was used to identify molecular markers linked to the *Glossy* gene (*BnaA.GL*) [31]. Three plants of each phenotypic class (normal and glossy plants) from the BC_3_ mapping population were randomly selected for constructing the normal bulk (NB) and glossy bulk (GB). The AFLP analysis and conversion of AFLP markers into SCAR markers were performed as previously described by Zeng et al. [53]. The IP and SSR marker development was performed as previously described by Xia et al. [39]. For primers used in mapping see Additional file 6.

A SNP polymorphism analysis was performed using SNP chip assay combined with BSA. WT, mutant, normal bulk (NB) and glossy bulk (GB) individuals were genotyped using the Illumina Brassica 60 K Beadchip developed by an international *Brassica* SNP array Consortium, which was led by AAFC (Agriculture and Agri-Food, Canada). Genotyping was performed according to the manufacturer’s recommendations using the Illumina iScan System (Illumina Inc., San Diego, CA).

Subsequently, a BC_3_F_2_ population consisting of more than 1200 individuals was generated, and 300 individuals with wax from this population were used. The data of these markers and individual phenotypes were analyzed with the MAP-MAKER/EXP 3.0 program [54]. All the markers linked to genes were used to compare the micro-colinearity of the regions flanking the genes with *B. rapa* and *Arabidopsis*, as indicated in previous reports [39].

### cDNA microarray assay

WT, *GL* mutant and DH line bulks based on phenotype were used. Three independent RNA samples for each were assayed and analyzed. Total RNA of 4-week-old leaves was extracted using the RNeasy Plant Mini Kit (Qiagen).

Approximately 500 ng total RNA was reverse transcribed using Message™ II aRNA Amplification Kit (Ambion) according to the manufacturer's protocol. A single dye (Cy5) was used for labeling (Kreatch diagnostics).

A total of 12 independent hybridizations were performed using the Combimatrix Brassica 90 K microarray produced at the NRC-PBI (National Research Council Plant Biotechnology Institute) (The Hybridization Custom Array™ 90 K Microarray: Protocol PTL020). Over 94,000 unique DNA probes were synthesized in situ using the patented Combimatrix virtual-flask technology. The semiconductor-based arrays consist of 29 row × 34 column spots. Data collection was performed as previously described by Zhu et al. [55].

R software was used to statistically analyze the microarray data. Data preprocessing and DEG detection were conducted with the bioconductor [56] packages limma [57] and RankProd [58], respectively. DEGs were selected with FDR < 0.05 as a threshold. The GO term classification of the DEGs was completed according to ATH_GO_GOSLIM from TAIR [59].

### Analysis of transcription levels by quantitative RT-PCR and semiquantitative RT-PCR

The primer pairs for the quantitative real-time RT-PCR and semiquantitative RT-PCR were specific for the genes and designed to cover an 80-200-bp region (Additional file 6). First-strand cDNAs were synthesized in a 20-μL reaction volume containing approximately 1 μg RNA using the PrimeScript™ 1st Strand cDNA Synthesis Kit (Takara) with oligo dT primers. BnACTIN was applied as an endogenous control for standardization for the real-time PCR templates, and total RNA was extracted from 6-week-old leaves. Quantitative RT-PCR was performed using SYBR Green Real-time PCR Master Mix (TOYOBO) with 0.8 μL of each primer (10 μM) and 1 μL of 1:100 diluted cDNA template in a 20-μl reaction mixture. The results from three biological replicates are shown.

For semiquantitative real-time PCR templates, 18S rRNA was applied as an endogenous control. Amplified PCR products (10 μL) were resolved on a 1% (w/v) agarose gel with 1× TBE running buffer.

### Toluidine blue (TB) test

Toluidine blue is a metachromatic dye that can bind to free anionic groups, such as carboxylate and phosphate. The TB test is able to evaluate water permeability due to surface deficiency, as described previously [33,60,61]. Leaves and stems from four-week-old plants were incubated in an aqueous solution of 0.05% (w/v) Toluidine blue for 2 min and then rinsed with water to remove excess TB from the leaf surface.

### Chlorophyll measurements

The leaves of 4-week-old plants were used for chlorophyll measurement. Approximately 2 g of each leaf sample was incubated on ice for 20 min and immersed in 40 mL of 80% ethanol in 50-mL aluminum foil-wrapped conical tubes at room temperature. Aliquots of 500 μl were removed from the solution every 20 min after the initial immersion. The amount of extracted chlorophyll was quantified by measuring the absorbance at 647 and 664 nm using a spectrophotometer, as described previously [62]. We calculated the concentration of total chlorophyll in the fresh leaf tissue using the following equation: total micromoles chlorophyll = 7.93(A664) + 19.53(A647). Chlorophyll efflux during each interval was expressed as a percentage of the chlorophyll over the total chlorophyll extracted after 24 h [62].

### Measurement of water loss

For the water loss rate analysis, 4-week-old leaves were excised and soaked in water for 60 min in the dark. The leaves were dried and weighed at the indicated time points at room temperature and dried in a 70°C oven overnight until the dry weight was constant. Total water was calculated as the fresh weight minus the dry weight after the heat treatment. The water loss at each interval was expressed as a percentage of the water loss over the total water [7,10].

### Availability of supporting data

Results for the cDNA microarray assays are available through ArrayExpress http://www.ebi.ac.uk/arrayexpress/ website under the accession number E-MEXP-3989.

The sequences of *BnCER1* in WT (*BnCER1.1*) and in *GL* mutant (*BnCER1.2*) are available in the NCBI GenBank under the accession numbers KF724897, KF72488, respectively.

## Abbreviations

CER: ECERIFERUM; VLCFA: Very long-chain fatty acid; DH: Double haploid; SEM: Scanning electron microscopy; TEM: transmission electron microscopy; TB: Toluidine Blue; TLC: Thin-layer chromatography; AFLP: Amplified fragment-length polymorphism; SCAR: Sequence-characterized amplified region; SNP: Single-nucleotide polymorphism; BSA: With bulk segregant analysis; IP: Intron polymorphism; SSR: Two single sequence repeat; BAC: Bacterial artificial chromosome; BCCP: Biotin carboxyl carrier protein; FATB: Fatty acyl-ACP thioesterases B; MAH1: Midchain alkane hydroxylase; WSD1: Bifunctional enzyme wax synthase.

## Competing interests

The authors have declared that no competing interests exist.

## Authors’ contributions

YP, JS and JZ designed and supervised the study, JT, TF and CM participated in its design. JG carried out the microarray data analysis. YG and LZ participated in the mapping. YP carried out the microarray chip hybrid analysis, biochemical and RT-PCR analysis. JW, PX and TL participated in the statistical analysis. BY participated in SNP chip assays. YP and JZ wrote the manuscript. All the authors discussed the results and contributed to the manuscript. All authors read and approved the final manuscript.

## Supplementary Material

Additional file 1 **Comparison of water permeability of leaf between WT and *GL *** **mutant.** Chlorophyll leaching assays (expressed as a percentage of total chlorophyll extracted after 24 h). The data represent means of mean values ± SE (n = 3). After 100 min of incubation with alcohol (80% w/v), mutant leaves had lost about 88% of their chlorophyll, while the WT only lost about 54%. Water loss assays (expressed as a percentage of total water loss after 24 h). The data represent means of mean values ± SE (n = 3). The leaf at 4 weeks post emergency excised and soaked in water for 60 min in the dark. They were dried and weighed per 60 min. Click here for file

Additional file 2 Explanations to microarray data. Click here for file

Additional file 3 **Functional classification of genes.** The number follows the term indicate the number of DEGs in this class. Functional classification of up-regulated genes in WT VS mutant. **A**. Functional classification of up-regulated genes in DHNB VS DHGB. **B**. Functional classification of up-regulated genes in both comparation. **C**. Functional classification of down-regulated genes in WT VS mutant. **D**. Functional classification of down-regulated genes in DHNB VS DHGB. Functional classification of down-regulated genes in both comparation. Click here for file

Additional file 4 **The major sequence differences between the WT and *GL *** **mutant.** Click here for file

Additional file 5 Most of the detected SNPs were located on chromosome N9. Click here for file

Additional file 6 Primers used in this study. Click here for file
