# Supplementary material for: A novel dominant glossy mutation causes suppression of wax biosynthesis pathway and deficiency of cuticular wax in Brassica napus
Source: BMC Plant Biol. 2013 Dec 14;13:215. doi: 10.1186/1471-2229-13-215 (PMC3881019; doi:10.1186/1471-2229-13-215)
Supplement: Additional file 1 — Comparison of water permeability of leaf between WT and GL mutant. Chlorophyll leaching assays (expressed as a percentage of total chlorophyll extracted after 24 h). The data represent means of mean values ± SE (n = 3). After 100 min of incubation with alcohol (80% w/v), mutant leaves had lost about 88% of their chlorophyll, while the WT only lost about 54%. Water loss assays (expressed as a percentage of total water loss after 24 h). The data represent means of mean values ± SE (n = 3). The leaf at 4 weeks post emergency excised and soaked in water for 60 min in the dark. They were dried and weighed per 60 min. [file 1471-2229-13-215-S1.docx]

**Additional file 1**

**Comparison of water permeability of leaf between WT and *GL* mutant**

1. Chlorophyll leaching assays (expressed as a percentage of total chlorophyll extracted after 24 h). The data represent means of mean values ±SE (n=3).

After 100 min of incubation with alcohol (80% w/v), mutant leaves had lost about 88% of their chlorophyll, while the WT only lost about 54 %.

1. Water loss assays (expressed as a percentage of total water loss after 24 h). The data represent means of mean values ±SE (n=3).

The leaf at 4 weeks post emergency excised and soaked in water for 60 min in the dark. They were dried and weighed per 60 min
