# Supplementary material for: A novel dominant glossy mutation causes suppression of wax biosynthesis pathway and deficiency of cuticular wax in Brassica napus
Source: BMC Plant Biol. 2013 Dec 14;13:215. doi: 10.1186/1471-2229-13-215 (PMC3881019; doi:10.1186/1471-2229-13-215)
Supplement: Additional file 3 — Functional classification of genes. The number follows the term indicate the number of DEGs in this class. Functional classification of up-regulated genes in WT VS mutant. A. Functional classification of up-regulated genes in DHNB VS DHGB. B. Functional classification of up-regulated genes in both comparation. C. Functional classification of down-regulated genes in WT VS mutant. D. Functional classification of down-regulated genes in DHNB VS DHGB. Functional classification of down-regulated genes in both comparation. [file 1471-2229-13-215-S3.docx]

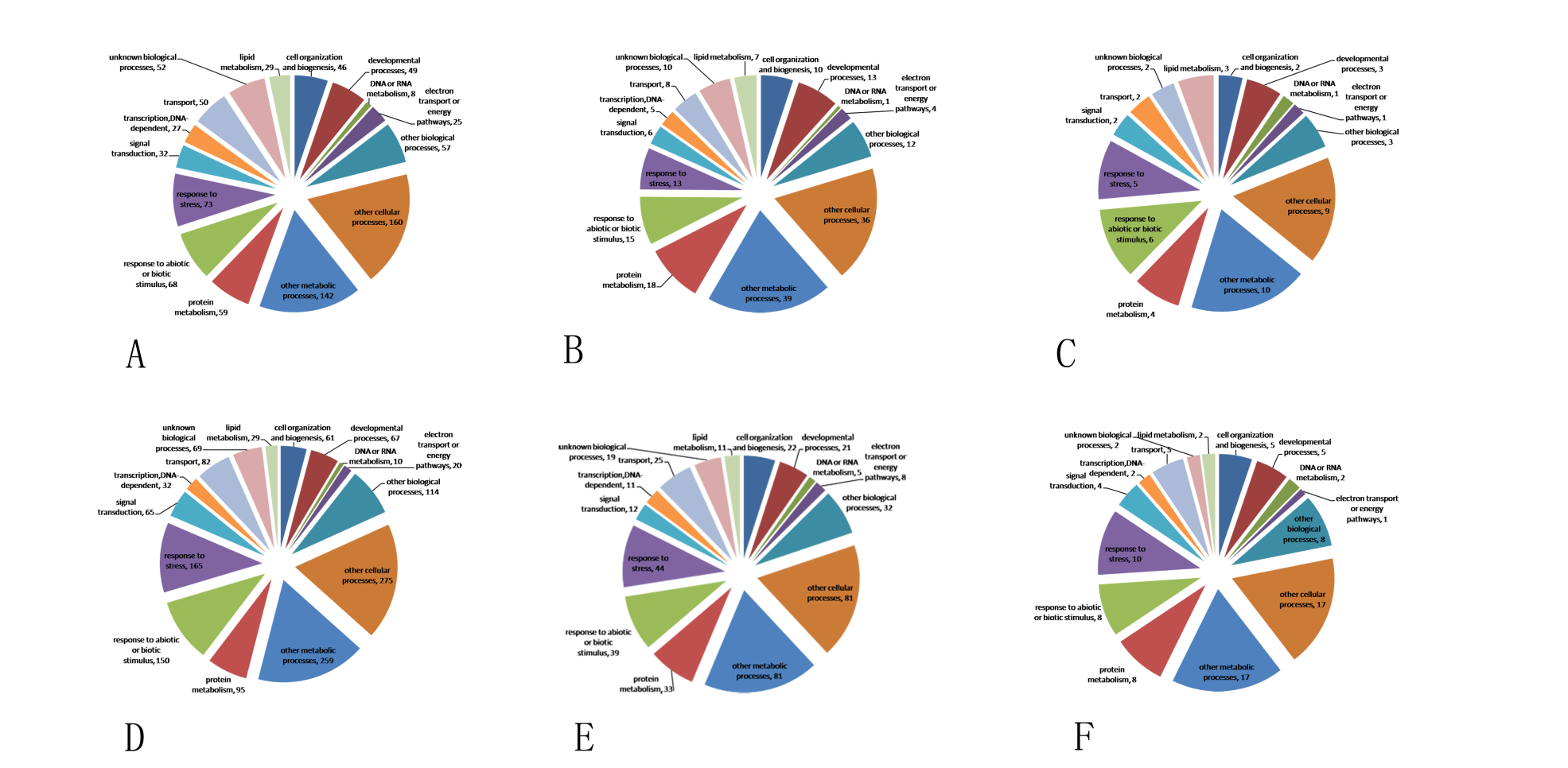


**Additional file 3 Functional classification of genes**

The number follows the term indicate the number of DEGs in this class.

1. Functional classification of up-regulated genes in WT VS mutant.
2. Functional classification of up-regulated genes in DHNB VS DHGB.
3. Functional classification of up-regulated genes in both comparation.
4. Functional classification of down-regulated genes in WT VS mutant.
5. Functional classification of down-regulated genes in DHNB VS DHGB.
6. Functional classification of down-regulated genes in both comparation.
