# Supplementary material for: A novel dominant glossy mutation causes suppression of wax biosynthesis pathway and deficiency of cuticular wax in Brassica napus
Source: BMC Plant Biol. 2013 Dec 14;13:215. doi: 10.1186/1471-2229-13-215 (PMC3881019; doi:10.1186/1471-2229-13-215)
Supplement: Additional file 4 — The major sequence differences between the WT and GL mutant. [file 1471-2229-13-215-S4.rtf]

  Additional file4 The major sequence differences between the WT and GL mutant                                                                  
                      20                 40                 60       
WT : ATGGCTACGAAACCAGGCATCCTCACCGATTGGCCTTGGACACCCCTTGGGAATTTCAAG :   60
GL : ATGGCTACGAAACCAGGCATCCTCACCGATTGGCCTTGGACACCCCTTGGGAATTTCAAG :   60
     ATGGCTACGAAACCAGGCATCCTCACCGATTGGCCTTGGACACCCCTTGGGAATTTCAAG       
                                                                        
                      80                100                120       
WT : GTGCATTCTTTTTTCTTTCTCTCTTTGGTTTGTCTATTTAATTATTATATATATTGTTCT :  120
GL : GTGCATTCTTTTTTCTTTCTCTCTTTGGTTTGTCTATTTAATTATTATATATATTGTTCT :  120
     GTGCATTCTTTTTTCTTTCTCTCTTTGGTTTGTCTATTTAATTATTATATATATTGTTCT       
                                                                        
                     140                160                180       
WT : ACTAAAAAACACTATTATGTAGTATATATATATATAAAAAACTCGTGCTTATGATCGAAT :  180
GL : ACTAAAAAACACTATTATGTAGTATATATATATATAAAAAACTCGTGCTTATGATCGAAT :  180
     ACTAAAAAACACTATTATGTAGTATATATATATATAAAAAACTCGTGCTTATGATCGAAT       
                                                                        
                     200                220                240       
WT : CCCGTTTCTGATGCATGATATGGTACGTGGAAACAATTAATACAATAGTACATAGTAATA :  240
GL : CCCGTTTCTGATGCATGATATGGTACGTGGAAACAATTAATACAATAGTACATAGTAATA :  240
     CCCGTTTCTGATGCATGATATGGTACGTGGAAACAATTAATACAATAGTACATAGTAATA       
                                                                        
                     260                280                300       
WT : GCACCATGGGCGGTCCATAGCACATACAAGTTTGTGACTGATGATCCGGTGGATCTCGGA :  300
GL : GCACCATGGGCGGTCCATAGCACATACAAGTTTGTGACTGATGATCCGGTGGATCTCGGA :  300
     GCACCATGGGCGGTCCATAGCACATACAAGTTTGTGACTGATGATCCGGTGGATCTCGGA       
                                                                        
                     320                340                360       
WT : TACTCCCTCGTATTGCCCTTCTTGCTCTTCAGAATTCTTCACAACCAGGTTTGGATCTCT :  360
GL : TACTCCCTCGTATTGCCCTTCTTGCTCTTCAGAATTCTTCACAACCAGGTTTGGATCTCT :  360
     TACTCCCTCGTATTGCCCTTCTTGCTCTTCAGAATTCTTCACAACCAGGTTTGGATCTCT       
                                                                        
                     380                400                420       
WT : CTTTCCCGTTACTATACAACCAAGGGAAAGAGACGCATCCTCGACAAAGGTATCGACTTC :  420
GL : CTTTCCCGTTACTATACAACCAAGGGAAAGAGACGCATCCTCGACAAAGGTATCGACTTC :  420
     CTTTCCCGTTACTATACAACCAAGGGAAAGAGACGCATCCTCGACAAAGGTATCGACTTC       
                                                                        
                     440                460                480       
WT : AATCAGGTCGACAGGGAGACCAACTGGTGTGTTTTTCACTCTGTTTGTCATTTTATGCAA :  480
GL : AATCAGGTCGACAGGGAGACCAACTGGTGTGTTTTTCACTCTGTTTGTCATTTTATGCAA :  480
     AATCAGGTCGACAGGGAGACCAACTGGTGTGTTTTTCACTCTGTTTGTCATTTTATGCAA       
                                                                        
                     500                520                540       
WT : CTGCGTCGCTTGTGCTTTATGTGAGCTAAGTATGTATTCTTGATATTTACGCGCGCAGGG :  540
GL : CTGCGTCGCTTGTGCTTTATGTGAGCTAAGTATGTATTCTTGATATTTACGCGCGCAGGG :  540
     CTGCGTCGCTTGTGCTTTATGTGAGCTAAGTATGTATTCTTGATATTTACGCGCGCAGGG       
                                                                        
                     560                580                600       
WT : ATGACCAAATATTGTTCAACGGATTGCTGTTCTATATAGGGATCATGCTGCTGCCGCAGG :  600
GL : ATGACCAAATATTGTTCAACGGATTGCTGTTCTATATAGGGATCATGCTGCTGCCGCAGG :  600
     ATGACCAAATATTGTTCAACGGATTGCTGTTCTATATAGGGATCATGCTGCTGCCGCAGG       
                                                                        
                     620                640                660       
WT : CAAAGCAACTTCCCTGGTGGAGGACAGACGGAGTGTTGATGGCTGCGATGCTTCATGCCG :  660
GL : CAAAGCAACTTCCCTGGTGGAGGACAGACGGAGTGTTGATGGCTGCGATGCTTCATGCCG :  660
     CAAAGCAACTTCCCTGGTGGAGGACAGACGGAGTGTTGATGGCTGCGATGCTTCATGCCG       
                                                                        
                     680                700                720       
WT : GGCCGGTGGAGTTCCTCTATTATTGGCTCCACAAAGCTCTTCACCACCACTTTCTTTACT :  720
GL : GGCCGGTGGAGTTCCTCTATTATTGGCTCCACAAAGCTCTTCACCACCACTTTCTTTACT :  720
     GGCCGGTGGAGTTCCTCTATTATTGGCTCCACAAAGCTCTTCACCACCACTTTCTTTACT       
                                                                        
                     740                760                780       
WT : CCCGCTACCATTCCCACCACCACTCCTCTATCGTCACTGAACCCATCACTTGTAACTCTT :  780
GL : CCCGCTACCATTCCCACCACCACTCCTCTATCGTCACTGAACCCATCACTTGTAACTCTT :  780
     CCCGCTACCATTCCCACCACCACTCCTCTATCGTCACTGAACCCATCACTTGTAACTCTT       
                                                                        
                     800                820                840       
WT : ATTTCTTATCTGTTATTATTTTTTTATCAAATTTACCTAAAATTTTCCACGTCCTAGTTT :  840
GL : ATTTCTTATCTGTTATTATTTTTTTATCAAATTTACCTAAAATTTTCCACGTCCTAGTTT :  840
     ATTTCTTATCTGTTATTATTTTTTTATCAAATTTACCTAAAATTTTCCACGTCCTAGTTT       
                                                                        
                     860                880                900       
WT : AGTAATATATATTTTTAATCATTATATTCAGCGGTGATACATCCATTCGCGGAGCATATA :  900
GL : AGTAATATATATTTTTAATCATTATATTCAGCGGTGATACATCCATTCGCGGAGCATATA :  900
     AGTAATATATATTTTTAATCATTATATTCAGCGGTGATACATCCATTCGCGGAGCATATA       
                                                                        
                     920                940                960       
WT : GCATACTTCATTCTTTTCGCCATACCATTGTTGACAACGTTGTTAACGAAAACGGCATCC :  960
GL : GCATACTTCATTCTTTTCGCCATACCATTGTTGACAACGTTGTTAACGAAAACGGCATCC :  960
     GCATACTTCATTCTTTTCGCCATACCATTGTTGACAACGTTGTTAACGAAAACGGCATCC       
                                                                        
                     980               1000               1020       
WT : ATAGCGTCGTTCTCCGGATATGTAATCTACATAGACTTCATGAACAACATGGGACACTGC : 1020
GL : ATAGCGTCGTTCTCCGGATATGTAATCTACATAGACTTCATGAACAACATGGGACACTGC : 1020
     ATAGCGTCGTTCTCCGGATATGTAATCTACATAGACTTCATGAACAACATGGGACACTGC       
                                                                        
                    1040               1060               1080       
WT : AACTTCGAGCTCGTCCCTAAGCGCCTTTTCCACCTCTTTCCTCCACTCAAGTACCTCTGC : 1080
GL : AACTTCGAGCTCGTCCCTAAGCGCCTTTTCCACCTCTTTCCTCCACTCAAGTACCTCTGC : 1080
     AACTTCGAGCTCGTCCCTAAGCGCCTTTTCCACCTCTTTCCTCCACTCAAGTACCTCTGC       
                                                                        
                    1100               1120               1140       
WT : TACACTCCCTCGTAAGTCCTTGCACTTTCGTACCATATATATATACATTTATTTCTGTCG : 1140
GL : TACACTCCCTCGTAAGTCCTTGCACTTTCGTACCATATATATATACATTTATTTCTGTCG : 1140
     TACACTCCCTCGTAAGTCCTTGCACTTTCGTACCATATATATATACATTTATTTCTGTCG       
                                                                        
                    1160               1180               1200       
WT : GCTATATATATGTCAAAATTTAGTACCAGTTTTTTTTCAAAACAATTTTGACTTACCTAT : 1200
GL : GCTATATATATGTCAAAATTTAGTACCAGTTTTTTTTCAAAACAATTTTGACTTACCTAT : 1200
     GCTATATATATGTCAAAATTTAGTACCAGTTTTTTTTCAAAACAATTTTGACTTACCTAT       
                                                                        
                    1220               1240               1260       
WT : GCATTAACCGGAAACCATTCTTTACATAAAAAAATACAAACAACGATTTTAATTATTATT : 1260
GL : GCATTAACCGGAAACCATTCTTTACATAAAAAAATACAAACAACTATTTTAATTATTATT : 1260
     GCATTAACCGGAAACCATTCTTTACATAAAAAAATACAAACAAC ATTTTAATTATTATT       
                                                                        
                    1280               1300               1320       
WT : ATTAATTTTTATCCGCATCACTTGCATTTTCTTTTCTCAAAAA-AAAAATATCACTTGCA : 1319
GL : ATTAATTTTTATCCGCATCACTTGCATTTTCTTTTCTCAAAAATAAAAATTTCACTTGCA : 1320
     ATTAATTTTTATCCGCATCACTTGCATTTTCTTTTCTCAAAAA AAAAAT TCACTTGCA       
                                                                        
                    1340               1360               1380       
WT : TTTGCATGTATACGTACATTCTCCTTTCATGTACAAAAAGGTGACCAGACATACTTGTAC : 1379
GL : TTTGCATGTATACGTACATTCTCCTTTCATGTACAAAAAGGTGACCAGACATACTTGTAC : 1380
     TTTGCATGTATACGTACATTCTCCTTTCATGTACAAAAAGGTGACCAGACATACTTGTAC       
                                                                        
                    1400               1420               1440       
WT : CCTTTTTTTTTGTTGTTAACTACCGAATACATGGACCACATAAAAATACAATATAATCTA : 1439
GL : CCTTTTTTTTTGTTGTTAACTACCGAATACATGGACCACATAAAAATACAATATAATCTA : 1440
     CCTTTTTTTTTGTTGTTAACTACCGAATACATGGACCACATAAAAATACAATATAATCTA       
                                                                        
                    1460               1480               1500       
WT : TATGCACATAATCCAATTTTCCACAATATGATGTATTTAGAGCCTATTTAACCAAACAGC : 1499
GL : TATGCACATAATCCAATTTTCCACAATATGATGTATTTAGAGCCTATTTAACCAAACAGC : 1500
     TATGCACATAATCCAATTTTCCACAATATGATGTATTTAGAGCCTATTTAACCAAACAGC       
                                                                        
                    1520               1540               1560       
WT : CTTAGAAAAGTAAATATCAAATTCATTAGGGTATATGGCTCTTCTAAAGTGTAGAGTTAT : 1559
GL : CTTAGAAAAGTAAATATCAAATTCATTAGGGTATATGGCTCTTCTAAAGTGTAGAGTTAT : 1560
     CTTAGAAAAGTAAATATCAAATTCATTAGGGTATATGGCTCTTCTAAAGTGTAGAGTTAT       
                                                                        
                    1580               1600               1620       
WT : TAACACAGCTTTGTGTTTTCCGTATATCTATAACGAGCGGAATGTGATAAAACACCCTTA : 1619
GL : TAACACAGCTTTGTGTTTTCCGTATATCTATAACGAGCGGAATGTGATAAAACACCCTTA : 1620
     TAACACAGCTTTGTGTTTTCCGTATATCTATAACGAGCGGAATGTGATAAAACACCCTTA       
                                                                        
                    1640               1660               1680       
WT : TTGTTGTAATATCCTTCTTTTGGATGGTAAAATGATCATAATGAGAGTTGCAGATTCCAC : 1679
GL : TTGTTGTAATATCCTTCTTTTGGATGGTAAAATGATCATAATGAGAGTTGCAGATTCCAC : 1680
     TTGTTGTAATATCCTTCTTTTGGATGGTAAAATGATCATAATGAGAGTTGCAGATTCCAC       
                                                                        
                    1700               1720               1740       
WT : TCGCTGCACCACACCCAATTCCGAACTAACTACTCCCTCTTCATGCCCTTGTATGACTAC : 1739
GL : TCGCTGCACCACACCCAATTCCGAACTAACTACTCCCTCTTCATGCCCTTGTATGACTAC : 1740
     TCGCTGCACCACACCCAATTCCGAACTAACTACTCCCTCTTCATGCCCTTGTATGACTAC       
                                                                        
                    1760               1780               1800       
WT : ATCTACGGCACAATGGATGAAACCTCGGATACATTGTATGAGAAATCTCTAGAAAGAGGA : 1799
GL : ATCTACGGCACAATGGATGAAACCTCGGATACATTGTATGAGAAATCTCTAGAAAGAGGA : 1800
     ATCTACGGCACAATGGATGAAACCTCGGATACATTGTATGAGAAATCTCTAGAAAGAGGA       
                                                                        
                    1820               1840               1860       
WT : GAAGATAGAGTGGACGTCGTGCACTTAACTCACCTGACGACTCCCGAGTCCATATACCAT : 1859
GL : GAAGATAGAGTGGACGTCGTGCACTTAACTCACCTGACGACTCCCGAGTCCATATACCAT : 1860
     GAAGATAGAGTGGACGTCGTGCACTTAACTCACCTGACGACTCCCGAGTCCATATACCAT       
                                                                        
                    1880               1900               1920       
WT : TTACGGATTGGCTTGGCTTCATTTGCCTCCTACCCCTTCTCTTATAGATGGTTCATGCGC : 1919
GL : TTACGGATTGGCTTGGCTTCATTTGCCTCCTACCCCTTCTCTTATAGATGGTTCATGCGC : 1920
     TTACGGATTGGCTTGGCTTCATTTGCCTCCTACCCCTTCTCTTATAGATGGTTCATGCGC       
                                                                        
                    1940               1960               1980       
WT : CTTTTGTGGCCTTTCACATCTCTCTCCATGCTCTTCACTCTCTTCTACGCCAGCCTCTTT : 1979
GL : CTTTTGTGGCCTTTCACATCTCTCTCCATGCTCTTCACTCTCTTCTACGCCAGCCTCTTT : 1980
     CTTTTGTGGCCTTTCACATCTCTCTCCATGCTCTTCACTCTCTTCTACGCCAGCCTCTTT       
                                                                        
                    2000               2020               2040       
WT : GTCTCTGAGAGAAACTCTTTCGAGAAGCTCAACTTGCAGTCTTGGATCATACCCAGATAT : 2039
GL : GTCTCTGAGAGAAACTCTTTCGAGAAGCTCAACTTGCAGTCTTGGATCATACCCAGATAT : 2040
     GTCTCTGAGAGAAACTCTTTCGAGAAGCTCAACTTGCAGTCTTGGATCATACCCAGATAT       
                                                                        
                    2060               2080               2100       
WT : AATCTGCAGGTCCTCATATTTTTCAGCTTATTTTTGACTCTTTTGTGAATTTACAACCTG : 2099
GL : AATCTGCAGGTCCTCATATTTTTCAGCTTATTTTTGACTCTTTTGTGAATTTACAACCTG : 2100
     AATCTGCAGGTCCTCATATTTTTCAGCTTATTTTTGACTCTTTTGTGAATTTACAACCTG       
                                                                        
                    2120               2140               2160       
WT : TATAAGTAGTTTAGGTTGGTTTACTGATGCACTGAGATCTAATGCATGCAGTACTTGTTA : 2159
GL : TATAAGTAGTTTAGGTTGGTTTACTGATGCACTGAGATCTAATGCATGCAGTACTTGTTA : 2160
     TATAAGTAGTTTAGGTTGGTTTACTGATGCACTGAGATCTAATGCATGCAGTACTTGTTA       
                                                                        
                    2180               2200               2220       
WT : AAATGGAGGAAAGACGCGATCAACAACATGATTGAGAAAGCGATACTGGAGGCAAATGAA : 2219
GL : AAATGGAGGAAAGACGCGATCAACAACATGATTGAGAAAGCGATACTGGAGGCAAATGAA : 2220
     AAATGGAGGAAAGACGCGATCAACAACATGATTGAGAAAGCGATACTGGAGGCAAATGAA       
                                                                        
                    2240               2260               2280       
WT : AAAGGAGTGAAGGTGCTTAGCCTGGGTCTCATGAACCAAGTAAAGAAACACGGTTCCACT : 2279
GL : AAAGGAGTGAAGGTGCTTAGCCTGGGTCTCATGAACCAAGTAAAGAAACACGGTTCCACT : 2280
     AAAGGAGTGAAGGTGCTTAGCCTGGGTCTCATGAACCAAGTAAAGAAACACGGTTCCACT       
                                                                        
                    2300               2320               2340       
WT : GATTTGGTTTTACATTGGTGTGAGATGTCATTTCATAATATTAAAACTGTGGTGGGTTGG : 2339
GL : GATTTGGTTTTACATTGGTGTGAGATGTCATTTCATAATATTAAAACTGTGGTGGGTTGG : 2340
     GATTTGGTTTTACATTGGTGTGAGATGTCATTTCATAATATTAAAACTGTGGTGGGTTGG       
                                                                        
                    2360               2380               2400       
WT : AACAGGGGGAGGAGCTAAACAGGAATGGAGAGGTGTATATTCACAAGCATCCAGAAATGA : 2399
GL : AACAGGGGGAGGAGCTAAACAGGAATGGAGAGGTGTATATTCACAAGCATCCAGAAATGA : 2400
     AACAGGGGGAGGAGCTAAACAGGAATGGAGAGGTGTATATTCACAAGCATCCAGAAATGA       
                                                                        
                    2420               2440               2460       
WT : AAGTAAGAGTGGTGGACGGCAGTAGATTAACAGCAGCCGTTGTGATAAATAGTCTACCCA : 2459
GL : AAGTAAGAGTGGTGGACGGCAGTAGATTAACAGCAGCCGTTGTGATAAATAGTCTACCCA : 2460
     AAGTAAGAGTGGTGGACGGCAGTAGATTAACAGCAGCCGTTGTGATAAATAGTCTACCCA       
                                                                        
                    2480               2500               2520       
WT : AATCAACGACAAAAATAGTGATGACAGGCAATCTCACAAAGGTGGCATACACCATCGCCT : 2519
GL : AATCAACGACAAAAATAGTGATGACAGGCAATCTCACAAAGGTGGCATACACCATCGCCT : 2520
     AATCAACGACAAAAATAGTGATGACAGGCAATCTCACAAAGGTGGCATACACCATCGCCT       
                                                                        
                    2540               2560               2580       
WT : CTGCTCTTTGCCAGAGAGGTGTTGAGGTTGTGTTTTTCTCCTCCGTTTCCCCCCTTTTTT : 2579
GL : CTGCTCTTTGCCAGAGAGGTGTTGAGGTTGTGTTTTTCTCCTCCGTTTCCCCCCTTTTTT : 2580
     CTGCTCTTTGCCAGAGAGGTGTTGAGGTTGTGTTTTTCTCCTCCGTTTCCCCCCTTTTTT       
                                                                        
                    2600               2620               2640       
WT : TTGCTTGTCATTGCCTAAAGTAAAAGACAAATGAGTTTGAATTCCATATGTCCATAGGAG : 2639
GL : TTGCTTGTCATTGCCTAAAGTAAAAGACAAATGAGTTTGAATTCCATATGTCCATAGGAG : 2640
     TTGCTTGTCATTGCCTAAAGTAAAAGACAAATGAGTTTGAATTCCATATGTCCATAGGAG       
                                                                        
                    2660               2680               2700       
WT : AAAGCTTAAGTCCGCACCTTCACTTGTCCAAGCTCTCTTAAGTTGTATTATATATCATAA : 2699
GL : AAAGCTTAAGTCCGCACCTTCACTTGTCCAAGCTCTCTTAAGTTGTATTATATATCATAA : 2700
     AAAGCTTAAGTCCGCACCTTCACTTGTCCAAGCTCTCTTAAGTTGTATTATATATCATAA       
                                                                        
                    2720               2740               2760       
WT : TTCATTTCATTTGCTTGTAAATGTTATCTGCAGGTCTTGACTCTATTACCTGAAGAGTAT : 2759
GL : TTCATTTCATTTGCTTGTAAATGTTATCTGCAGGTCTTGACTCTATTACCTGAAGAGTAT : 2760
     TTCATTTCATTTGCTTGTAAATGTTATCTGCAGGTCTTGACTCTATTACCTGAAGAGTAT       
                                                                        
                    2780               2800               2820       
WT : GAGAAACTAAGTTCATTTGTTCCAAAAGAATGCAGAGACCGTTTGATCCTTTTAACCTCT : 2819
GL : GAGAAACTAAGTTCATTTGTTCCAAAAGAATGCAGAGACCGTTTGATCCTTTTAACCTCT : 2820
     GAGAAACTAAGTTCATTTGTTCCAAAAGAATGCAGAGACCGTTTGATCCTTTTAACCTCT       
                                                                        
                    2840               2860               2880       
WT : GAAACACTCGCATCAAACAAGGTAATTAAGTATACAATAAAAGTTGGAGAACAATCGAAG : 2879
GL : GAAACACTCGCATCAAACAAGGTAATTAAGTATACAATAAAAGTTGGAGAACAATCGAAG : 2880
     GAAACACTCGCATCAAACAAGGTAATTAAGTATACAATAAAAGTTGGAGAACAATCGAAG       
                                                                        
                    2900               2920               2940       
WT : AGCATACTAAACTAATGAATTTTGGTTGAAGGTATGGCTGATGGGAGAAGGAACAACGAG : 2939
GL : AGCATACTAAACTAATGAATTTTGGTTGAAGGTATGGCTGATGGGAGAAGGAACAACGAG : 2940
     AGCATACTAAACTAATGAATTTTGGTTGAAGGTATGGCTGATGGGAGAAGGAACAACGAG       
                                                                        
                    2960               2980               3000       
WT : AGAGGAGCAGGAAATGGCCACAAAAGGAACGTTATTTATCCCATTCTCACAGTTCCCCCT : 2999
GL : AGAGGAGCAGGAAATGGCCACAAAAGGAACGTTATTTATCCCATTCTCACAGTTCCCCCT : 3000
     AGAGGAGCAGGAAATGGCCACAAAAGGAACGTTATTTATCCCATTCTCACAGTTCCCCCT       
                                                                        
                    3020               3040               3060       
WT : CAAGCAGTTACGAAGAGATTGTATCTATCATACTCCACCGGCTTTAATAATTCCAAAATC : 3059
GL : CAAGCAGTTACGAAGAGATTGTATCTATCATACTCCACCGGCTTTAATAATTCCAAAATC : 3060
     CAAGCAGTTACGAAGAGATTGTATCTATCATACTCCACCGGCTTTAATAATTCCAAAATC       
                                                                        
                    3080               3100               3120       
WT : TCTGGTCAATATCCACTCCTGTGAGGTATGTATAAAAAGCTATCCAATCCAATTAAAAGT : 3119
GL : TCTGGTCAATATCCACTCCTGTGAGGTATGTATAAAAAGCTATCCAATCCAATTAAAAGT : 3120
     TCTGGTCAATATCCACTCCTGTGAGGTATGTATAAAAAGCTATCCAATCCAATTAAAAGT       
                                                                        
                    3140               3160               3180       
WT : TTTTGCCGATATGCTAAGTAACTAAATAAAACACATATGCAGAACTGGTTACCGAGAAAG : 3179
GL : TTTTGCCGATATGCTAAGTAACTAAATAAAACACATATGCAGAACTGGTTACCGAGAAAG : 3180
     TTTTGCCGATATGCTAAGTAACTAAATAAAACACATATGCAGAACTGGTTACCGAGAAAG       
                                                                        
                    3200               3220               3240       
WT : GCGATGAGTGCAACTAGAGTGGCTGGCATATTGCACGCCTTAGAAGGATGGGAAACGCAT : 3239
GL : GCGATGAGTGCAACTAGAGTGGCTGGCATATTGCACGCCTTAGAAGGATGGGAAACGCAT : 3240
     GCGATGAGTGCAACTAGAGTGGCTGGCATATTGCACGCCTTAGAAGGATGGGAAACGCAT       
                                                                        
                    3260               3280               3300       
WT : GAGTGCGGCACATCCAATATTCTTCTCTCAGATTTGGACCAAGTGTGGGAAGCTTGTCTC : 3299
GL : GAGTGCGGCACATCCAATATTCTTCTCTCAGATTTGGACCAAGTGTGGGAAGCTTGTCTC : 3300
     GAGTGCGGCACATCCAATATTCTTCTCTCAGATTTGGACCAAGTGTGGGAAGCTTGTCTC       
                                                               
                    3320               3340                 
WT : AGCCACGGCTTCCAGCCTCTGCTGCTTCCACATCACTTCCAATACCCCTGA : 3350
GL : AGCCACGGCTTCCAGCCTCTGCTGCTTCCACATCACTTCCAATACCCCTGA : 3351
     AGCCACGGCTTCCAGCCTCTGCTGCTTCCACATCACTTCCAATACCCCTGA       
